# Strengthening Newborn Nutrition Through Establishment of the First Human Milk Bank in Vietnam

**DOI:** 10.1177/0890334420948448

**Published:** 2020-08-24

**Authors:** Kimberly Mansen, Tuan T. Nguyen, Nga Q. Nguyen, Chung T. Do, Hoang T. Tran, Nga T. Nguyen, Roger Mathisen, Vinh D. Nguyen, Yen T. K. Ngo, Kiersten Israel-Ballard

**Affiliations:** 1 11006 PATH, Seattle, WA, USA; 2 1311 Alive & Thrive Southeast Asia, FHI 360, Hanoi, Vietnam; 3 PATH, Hanoi, Vietnam; 4 Alive & Thrive Southeast Asia, FHI 360, Danang, Vietnam; 5 Danang Hospital for Women and Children, Danang, Vietnam; 6 Maternal and Child Health Department, Vietnam Ministry of Health, Hanoi, Vietnam; 7 Danang Department of Health, Danang, Vietnam

**Keywords:** breastfeeding, milk banking, milk bank, human milk, lactation

## Background

Reduction of newborn mortality through improved newborn care is a global health priority. Each year, 15 million infants are born prematurely (Chawanpaiboon et al., 2019), leading to 1 million deaths due to complications associated with prematurity (Liu et al., 2016), the leading cause of neonatal mortality globally. Sustainable Development Goal Target 3.2 (UNICEF, 2017) calls for countries to reduce neonatal mortality from 19/1,000 live births in 2016 to 12/1,000 by 2030. However, many countries are not on target to achieve this (United Nations, 2020). Through improved global recognition and alignment of newborn care practices, including *Every Newborn Action Plan* (World Health Organization [WHO] & United Nations Children’s Fund [UNICEF], 2014) and *Early Essential Newborn Care* ([EENC]; WHO, 2018), facilities worldwide are seeking meaningful, sustainable, and cost-effective models for improving newborn health.

Breastfeeding is one of the most powerful interventions to save infant lives globally (Victora et al., 2016). When breastfeeding or provision of mother’s own milk is not an option or is not available in sufficient supply for low-birthweight infants, due to maternal morbidity or mortality, abandonment or separation, or maternal milk still coming to volume, the World Health Organization (2011) recommends the provision of pasteurized donor human milk (PDHM) from a human milk bank (HMB), as superior to formula. A HMB is defined as a service established to recruit human milk donors, collect donated milk, and then process, screen, store, and distribute the milk to meet infants’ specific needs for optimal health (PATH, 2019a). Provision of donor human milk (DHM) compared to formula improves health outcomes (particularly through the prevention of necrotizing enterocolitis) among vulnerable neonates, especially those preterm and low birth weight (Arslanoglu et al., 2013; Boyd et al., 2007; Cacho et al., 2017). Researchers have demonstrated that implementation models which achieve the integration of a HMB into systems for protecting, promoting, and supporting breastfeeding are most effective (Arnold, 2006; Arslanoglu et al., 2013, DeMarchis et al., 2017; Israel-Ballard, 2018). Yet, global guidance and standards about the implementation of HMB systems is limited. Recommendations for best practices on integration of HMB systems into high-level neonatal care facilities to protect, promote, and support optimal breastfeeding practices are vitally needed to ensure that mothers receive lactation support and that PDHM is not used inappropriately (PATH, 2019b).

Although nearly 700 HMBs exist globally, the majority are in Europe; South, Central and North America; and some countries in Asia (PATH, 2019a). Most low and middle-income countries (LMIC), settings with a high burden of neonatal morbidity or mortality, do not have even one HMB and guidance specific to adapting HMB systems to LMIC settings is limited (PATH, 2019a). This manuscript describes the successful experience of establishing the first HMB in Vietnam and the lessons learned that could be applied to a regional expansion.

Vietnam is an optimal location for establishing an integrated HMB. It is a country in Southeast Asia with a population of 95 million. Each year, out of 1.6 million births, 19,000 infants die within the first 28 days of life (41% due to prematurity), which represents 70% of the country’s infant deaths and 55% of deaths under 5 years of age (UNICEF, 2017). Vietnam faces challenges with achieving optimal infant feeding: 26.5% of infants achieve early initiation of breastfeeding, and 24.3% of infants under 6 months are exclusively breastfed (UNICEF, 2017). In Vietnam, formula has been used in the neonatal unit in some instances for more than 50% of admitted infants (Tran et al., 2018). Vietnam has prioritized neonatal health and nutrition, and implemented multiple initiatives, including the WHO and UNICEF action plan initiated in 2014 to increase the prevalence of breastfeeding (i.e., enactment of Decree 100—a legal document—to regulate trading and use of nutritional products for infants, as well as feeding bottles and dummies), strengthen Baby-Friendly hospitals, and ensure Early Essential Newborn Care (EENC) practices (Vietnam Government, 2014; World Health Organization, 2014, 2015). Vietnam extended paid maternity leave to 6 months (Vietnam National Assembly, 2012a) and applied strong regulation on trading in and use of manufactured nutrition products for infants, feeding bottles, and teats (Vietnam Government, 2014; Vietnam National Assembly, 2012b). In this context, Vietnam sought to establish the country’s first human milk bank (HMB), as part of its comprehensive strategy to ensure all infants receive human milk and to improve newborn care.

**Key messages**

- Donor human milk from a human milk bank is recommended for low birthweight and premature infants when mother’s own milk is not available.
- A systematic, phased approach is an effective mechanism for establishing safe, quality, and locally appropriate human milk banking systems and to ensure that provision of donor human milk is integrated into clinical use.
- Ownership by the hospital implementing a human milk bank, complimented with local and national support, is necessary to create a human milk banking system to support those most in need.
- The systematic and stepwise approach used for implementing the first human milk bank in Vietnam serves as a model for scaling up where systems did not previously exist.

## The Development and Establishment of the First Human Milk Bank in Vietnam

The Danang Hospital for Women and Children (DNHWC), designated as one of the three WHO Centers of Excellence for EENC (Nguyen et al., 2018), was selected as the site for the first HMB in Vietnam. The DNHWC is a referral hospital responsible for technical support and the supervision of district hospitals in Danang, and in other provinces in central Vietnam. The Danang City Department of Health has a history of strong breastfeeding promotion, including the launch of provincial franchise services to provide counseling services on maternal, infant, and young child nutrition in the region, supported by Alive & Thrive, a global nutrition initiative (https://www.fhi360.org/projects/alive-thrive; Baker et al., 2013). Each year, there are 13,000–15,000 births at the DNHWC, with 350,000 outpatient visits and inpatient treatment cases, and over 30,000 women and 50,000 pediatric patients (Nguyen et al., 2018). The hospital’s neonatal unit provides treatment each day for approximately 120 babies with low birth weight, premature birth, or illnesses (Nguyen et al., 2018; Tran et al., 2015). Providing the best health services for women and children is the main objective of the hospital, including breastfeeding promotion and support. The neonatal unit applies Kangaroo Mother Care (KMC) guidelines for preterm and low birth weight babies, and complies with Decree 100. The staff also support capacity building activities related to KMC, EENC, and Integrated Management of Childhood Illness for staff in DNHWC, and other hospitals in Vietnam, as well as in other countries (Nguyen et al., 2018).

To detail the process involved in establishing the first HMB in Vietnam, we reviewed project documents, monitoring system reports, and project progress reports to capture activities performed, modifications made, and lessons learned throughout the project. We also reviewed findings from the facility assessment, formative assessment, baseline and other feeding assessments, and the costing assessment, along with the updates made to the standard operating procedures. We detail the early implementation of this project from November 2015 through April 2017. The results represented here are the summarized actions that were pivotal for the successful implementation and integration of the first HMB in Vietnam.

**Figure 1:**
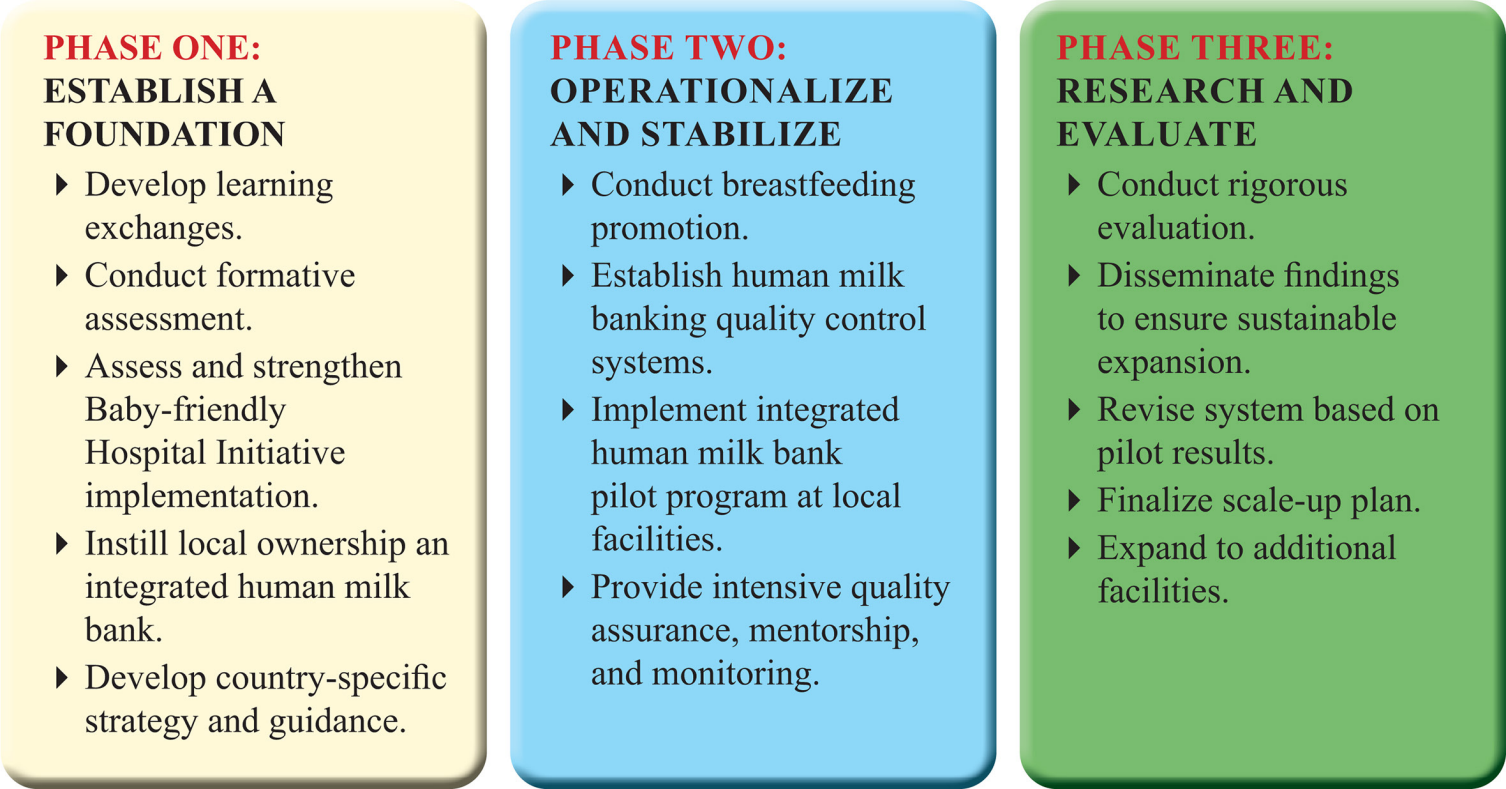
A Phased Approach for Establishing a New Human Milk Bank System. *Note*. This stepwise approach reveals the essential activities for establishing an integrated, sustainable human milk bank system in a new setting.

The implementation process involved establishing a multi-stakeholder partnership among the Ministry of Health (MoH), Danang City Department of Health, DNHWC, Alive & Thrive, and PATH, a global team of innovators working to eliminate health inequities (https://www.path.org). This team employed an established global model for a multi-phase, systematic approach to integrate a HMB with newborn care and nutrition programming, by instilling ownership, building technical competency, and ensuring sustainability and safety (Figure 1; PATH, 2019a). This built upon the learnings from past HMB implementations of PATH’s Mother Baby Friendly Initiative Plus model globally, including in South Africa and India (PATH, 2019a). Comprehensive implementation involved three key components: building local ownership; implementation of a safe, sustainable, and data-driven system; and ongoing quality improvement. Building upon the established global multiphase model, we further expanded to four critical phases for establishing a sustainable HMB model specifically in Vietnam: 1) induction and awareness; 2) establishment and technical competency; 3) operation and stabilization; and 4) expansion of model and sharing results (Table 1).

**Table 1:** Critical Adapted Phases for Establishing a Sustainable Human Milk Bank Model in Vietnam.

Induction and awareness (November 2015–June 2016)
HMB project initiated and funding secured
Approval obtained from the Ministry of Health and Danang People’s Committee
Awareness meetings held with key technical and policy leaders
Stakeholders met and agreed on the implementation plan
HMB learning exchange in Glasgow, Scotland
Project Advisory Committee and HMB multi-disciplinary team established
Facility assessment completed
Formative assessment completed
Establishment and technical competency (June 2016–January 2017)
Breastfeeding promotion, protection, and support components strengthened
HMB facility upgraded and equipment purchased
HACCP training conducted
HACCP plan developed
HMB guidelines and SOPs developed
Training on SOPs conducted
Digital information management system constructed
Baseline feeding study conducted
Costing assessment for establishment of HMB conducted
Operation and stabilization (February 2017 to date)
HMB launched
Demand generation activities (events, one-on-one contact, mass media, social network) conducted
Refresher training on SOPs conducted
HACCP refresher training conducted
Supportive supervision training conducted
Ongoing mentorship for quality improvement implemented
Endline evaluation of HMB (feeding study and assessing barriers and facilitators to implementation) conducted
Costing assessment for operation of HMB conducted
Expansion and sharing (May 2017 to date)
A Resource Toolkit for Establishing and Integrating Human Milk Bank Programs—A Global Implementation Framework Version 2.0 disseminated
Knowledge exchange supported
Technical assistance provided to additional hospitals in Vietnam and in Southeast Asia
HMB service agreements initiated with additional hospitals in Vietnam

*Note*. HMB = human milk bank. HACCP = Hazard Analysis and Critical Control Point. SOPs = standard operating procedures.

### Induction and Awareness (November 2015–June 2016)

#### Building Local Ownership

The initial phase entailed building ownership and awareness among key stakeholders. A stakeholder meeting was convened by Alive & Thrive and PATH in January 2016 to raise awareness among participants from the Maternal and Child Health Department and the Legislation Department from the MoH, Danang City Department of Health, UNICEF, an international HMB specialist, hospital leaders, social media breastfeeding advocates, and media. The chief neonatologist of the DNHWC, who also serves as the Vice Director of the hospital, served as a local champion, and built momentum with key stakeholders at DNHWC and with the Danang City Department of Health. A Project Advisory Committee, comprised of 10 leaders and staff of the MoH, Danang City Department of Health, Alive & Thrive, and PATH, was established to oversee the HMB implementation. The Project Advisory Committee held quarterly meetings throughout implementation, to agree on local needs and provide guidance. In addition to the Project Advisory Committee, a DNHWC multi-disciplinary, hospital based HMB team was established for integrating, establishing, and operating the HMB. It included experts in neonatology, nursing, microbiology, infection control, human milk processing, lactation support, hospital quality control, and midwifery.

#### Learning Exchange

Due to lack of established mechanisms for global learning or guidance from the WHO about establishing a HMB, the team relied on guidance provided voluntarily by the HMB staff in Glasgow, Scotland. To build technical competency, MoH, DNHWC, Alive & Thrive, and PATH had a 4-day learning exchange with the national Greater Glasgow and Clyde Donor Milk Bank in Glasgow. All HMB processes were shared, including all aspects of quality control regarding collection, screening, storage, and pasteurization, as well as the prioritization and appropriate use of PDHM in the neonate unit, donor recruitment, and the provision of lactation support (including early initiation of breastfeeding or milk expression and frequent expression for building maternal milk supply). Additional operational aspects, including staffing structure, costing, and the sustainability of the national service were also reviewed. After this learning exchange, Scotland HMB technical experts continued providing volunteer mentorship through remote and follow-up visits for on-site support.

#### Facility Assessment to Evaluate Readiness

A hospital assessment was performed by an international HMB expert from the United Kingdom and PATH to determine the readiness, barriers, and facilitators for optimal HMB implementation. Given that no global guidance existed and that this was the first HMB in Vietnam, an HMB expert with experience operating a human milk bank and feeding vulnerable infants was best suited to assess readiness, identify recommended locations, and observe the steps needed to establish a safe and quality system. The assessment included a site visit, review of medical records, and interviews with staff from the neonatal unit and postnatal ward, as well as observations of neonatal feeding methods. Maternal lactation was observed to be supported through access to and the support of trained nursing staff for encouraging breastfeeding and supporting expression, access to a communal breast pump, and posters with key breastfeeding support messages. The hospital assessment identified the strategic placement of the HMB within proximity to the public entrance, with proximal access to the neonatal unit. A review of the neonatal feeding records revealed that a more in-depth feeding assessment was needed to serve as a baseline for accurately assessing the enhancement of support for mother’s own milk and the accurate demand for PDHM.

#### Formative Assessment to Inform Communication Tools

A formative assessment was conducted to: (1) understand the perceptions and attitudes of mothers, hospital leadership, and health care providers towards establishing an HMB in Danang; (2) identify the barriers and facilitators influencing donation and use of donated human milk, including wet nursing; and (3) design a behavior change communication and demand generation strategy for acceptance of the services and to enable donation and use of safe PDHM. Focus group discussions were held with caretakers of potential recipients of PDHM, potential donors of DHM, and influencers, including grandmothers and partners. Technical experts, including health care providers and breastfeeding advocates were also interviewed.

Emerging key themes from the formative assessment included agreement on the need for PDHM, existence of milk sharing and wet nursing practices in the facility setting, and the perception that the donation of human milk was a humanitarian gesture and payment would not be required. HMB safety, hygiene, and the appropriate use of PDHM were the most pressing concerns. Based on the assessments, the HMB team developed a social and behavior change communication plan. A creative media agency assisted to develop, test, and produce various promotional products, including the HMB logo, video clip, posters, leaflets, donor identification/appreciation card, mascot for demand-generation activities, and recognition gifts for donors, guests, and volunteers. Social media channels (website, Facebook fan page) and demand-generation events were effectively used to raise awareness about the HMB and generate supply and demand. The HMB logo and slogan “sharing mother’s milk, sharing love” became the key theme throughout. An informative short video clip on HMB processes and safety was developed and aired on local television stations, HMB websites, and YouTube.

### Establishment and Technical Competency (June 2016–January 2017)

#### Facility Upgrade and Procurement of Equipment

Lactation support was an essential component of the comprehensive HMB model; therefore, space was allocated for a reception room for mothers interested in donating or needing lactation support. Facility requirements were established in alignment with local food safety guidelines for food production and business facilities. Multiple international human milk banking experts guided the HMB team and provided feedback based on their experiences. The DNHWC facilities’ director, along with members of the HMB team, supervised the construction of the HMB facility. For the procurement of necessary equipment and supplies, the Project Advisory Committee and HMB team, facilitated by PATH and Alive & Thrive, determined the optimal equipment and imported it. Licenses were obtained and the equipment was properly installed. Lacking reliable domestic suppliers, the human milk automated pasteurizer, containers for pasteurization, freezers, and laminar hood flow cabinet were imported. International equipment manufacturers provided guidance on the maintenance needs of the equipment, to be monitored locally.

#### Hazard Analysis and Critical Control Point (HACCP) Training to Inform Standard Operating Procedures (SOPs)

PATH, with support from a local certified quality control consultant, trained the multidisciplinary HMB team on the HACCP process, a quality assurance planning process for food systems, adapted to the HMB context (Arslanoglu et al., 2010; Hartmann, 2017; PATH, 2013, 2016). The HACCP workshop and information from the learning exchange led to a final HACCP plan, which served as a best practice for determining locally appropriate procedures, and revealed gaps where systems strengthening would be required for optimal quality and safety.

#### Development of Guidelines, SOPs, and Training Materials

**Figure 2:**
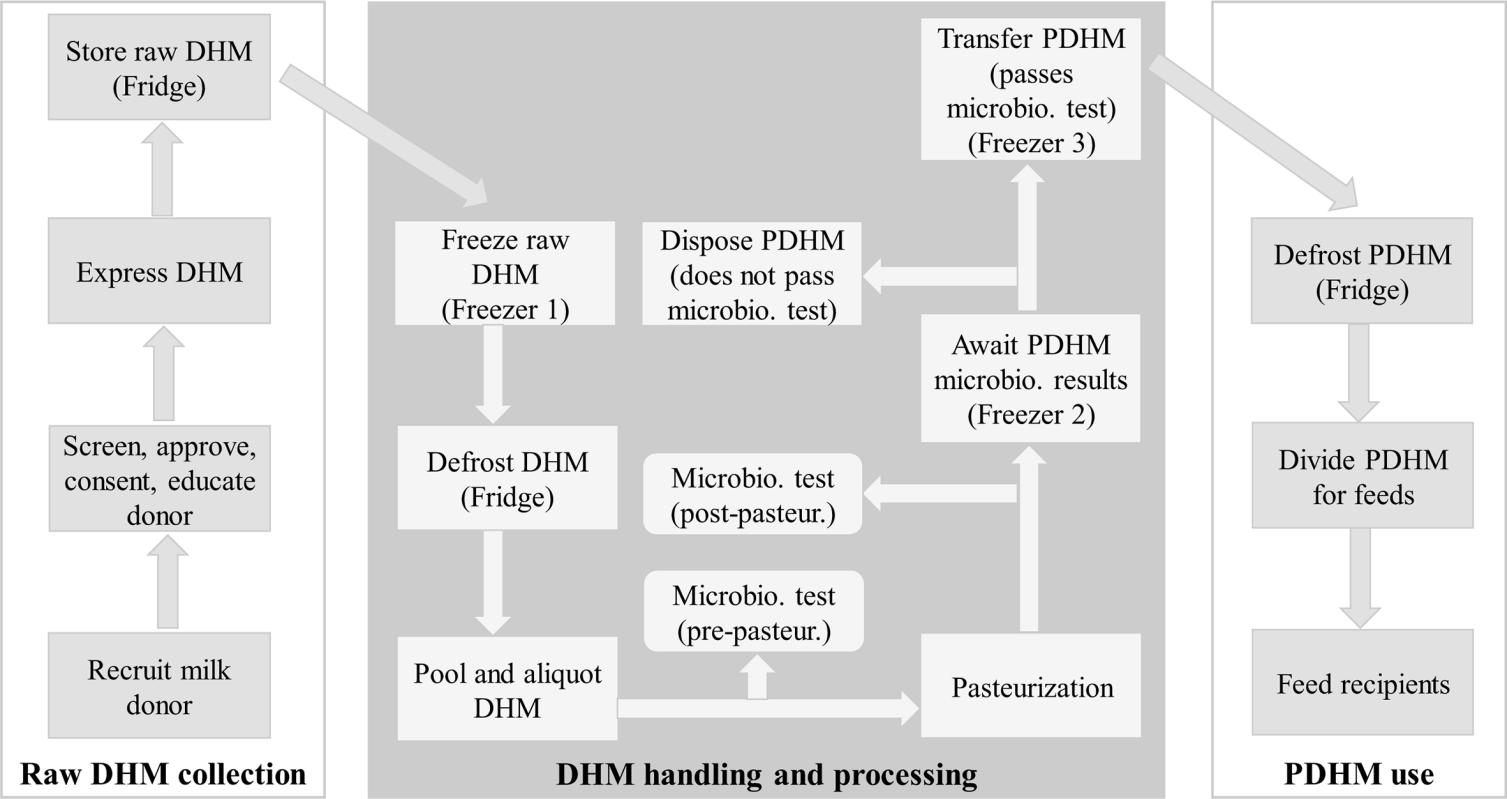
Operation Diagram of the Human Milk Bank at Danang Hospital for Women and Children. *Note*. This diagram explains the process that donor human milk follows throughout the human milk bank pathway from donor to recipient to maintain safety and quality. DHM = donor human milk; microbio. = microbiological; pasteur. = pasteurization; PDHM = pasteurized donor human milk.

Incorporating learnings from guidelines from other countries, the HMB team, with the support from the Project Advisory Committee, developed key guidelines and SOPs as a part of the process to ensure consistency and safety in practice (Centre for Clinical Practice at NICE, 2010; Human Milk Banking Association of North America, 2011, 2018; Philippines Department of Health, 2011). Every step in the HMB process (Figure 2) was detailed in three manuals of operation and 22 SOPs. The manuals and SOPs cover various components of HMB practices, including donor recruitment, donor screening, milk expression and handling, storage, pasteurization, microbial testing, and the distribution of PDHM to the neonatal unit (Table 2). Training materials were developed, targeting nursing staff from the newborn care and postnatal wards, nutrition and lactation support staff, microbiologists, and infection control and quality assurance staff, along with key leadership across hospital departments.

**Table 2:** List of Manuals of Operation and Standard Operating Procedures.

Manuals and Procedures	Location Where Applied			
	HMB	NU	PW	QA
Manuals of operation (MOP)				
Donor recruitment	√	√	√	
Donor education	√	√	√	
Feeding the recipient		√	√	
Standard operating procedures (SOP)				
Developing and approving a SOP	√	√	√	√
Regulations on recording documentation of donor milk	√	√	√	
Regulations on labeling donor milk	√			
HMB donor screening	√			
HMB donor selection and approval	√			
Hand washing and HMB staff’s health status condition	√			
Sending containers to HMB donor	√			
Milk storing	√	√	√	
Transport of donor milk	√			
Pasteurization	√			
Donor milk screening	√			
Donor milk testing	√			
Estimating need and ordering DHM		√	√	
Recipient prioritization and consent		√	√	
Defrosting, aliquoting	√			
Washing of HMB containers and tools	√			
Periodic maintenance of HMB facility and equipment	√			
Cleaning HMB facilities	√			
Internal monitoring and supervision				√
Detecting deviation and implementing corrective actions	√			
Pasteurizer operation and maintenance	√			
Training staff working on HMB related tasks	√			

*Note*. HMB = human milk bank; MOP = Manuals of operation; NU = Neonatal Unit/ Neonatal Intensive Care Unit; PW = Postnatal Ward; QA = Quality Assurance Unit; SOP = Standard operating procedures.

#### Training and Coaching on HMB-Related Topics

Most hospital staff, including all of those with a role in feeding neonates, were trained on HMB-related topics (e.g., donor recruitment and donor education; breastfeeding counseling and support; safe collection, handling and transporting of raw DHM; pasteurization process at the HMB; and indications and appropriate use of PDHM). In addition, HMB staff were provided with comprehensive training about the pasteurization process, taking samples of raw and pasteurized DHM for microbiological testing, and the use of the digital information system for tracking processes from donor recruitment to the use of PDHM.

#### Digital Information System to Optimize Operations

A digitized HMB monitoring system was developed to align with the guidelines and SOPs. It provided real-time data to (1) optimize the operation of the HMB; (2) ensure that all HMB activities follow standardized protocols; and (3) serve as a track and trace system for PDHM. The track and trace system included demand generation, donor management, milk processing, milk distribution, recipient management, PDHM usage, container tracking, and reporting. The electronic system helped to reduce paper-based monitoring forms, except those needed for legal status (e.g., informed consent, pasteurization procedure, testing results, and approval of PDHM use). This system also assisted milk tracking and tracing based on donor identification barcodes and container barcodes, facilitating rapid track and trace capability in the potential event of a recall of PDHM. The real-time data automatically generated reports and charts.

#### Baseline–Endline Feeding Assessments

Baseline and endline assessments were performed to determine the changes in feeding behaviors, and knowledge, attitudes, and practices of mother-baby dyads in the neonatal and post-natal departments at DNHWC before and 8 months after the launching of the HMB. Results will be presented in a subsequent manuscript.

#### Ensuring Sustainability: A Costing Assessment

A costing assessment performed by a team from Hanoi University of Public Health reviewed start up and recurring costing data from secondary sources, including financial reports and observations, and interviews to estimate the costs of PDHM and for sustaining the HMB. Publication of the findings of the costing study are underway.

### Operation and Stabilization (February 2017–April 2019)

The HMB officially launched in February 2017, marked by the collection, processing and storing, and allocation of PDHM to the neonatal unit. An official opening ceremony was held with a high level of visibility to showcase the importance of an HMB in saving newborn lives throughout Vietnam. Demand generation activities were subsequently conducted through events, one-on-one engagements, mass media, and social networking to advocate for the use of PDHM, the recruitment of donors, and the promotion of breastfeeding.

The HMB integrated into associated hospital services—neonatal and postnatal care, and lactation support activities—and improved the access to and intake of human milk for infants cared for at the DNHWC. During the 2 years of implementation (from February 2017 to April 2019), 755 mothers were approached for donor recruitment. Of these, 397 agreed to become donors; 314 of these passed screening tests and were trained with needed skills for safe donation, and subsequently donated milk to the HMB (Table 3). The donors had a mean age of 28 years; 60% resided in Danang City, 80% gave birth at DNHWC, and 50% donated during their child’s admission (Table 3). In total, the HMB collected 4,400 L of donor milk (Table 4). During the first 6 months of operation, 60% of the donations passed pre- and post-pasteurization screening, which later increased to approximately 80% in 2018. The total PDHM provided to the neonatal and postnatal wards during the first year of operation was 3,139 liters, of which 65% was administered within the neonatal unit (Table 4). The HMB served 8,071 vulnerable infants (~35% in the neonatal unit), who would otherwise have received human milk substitutes as mother’s own milk was insufficient or not available (Table 5). Average duration for the use of PDHM in the neonatal unit and postnatal ward was 4 days and 1 day, respectively (Table 5).

**Table 3:** Recruiting, Screening, and Managing Donor Mothers at the Human Milk Bank at Danang Hospital for Women and Children.

Activities	Location		
	*N*	In Hospital *n* (%)	Out of Hospital^a^ *n* (%)
Group counseling sessions for demand generation (≤ 10 mothers)	33	33 (100)	0 (0)
Demand generation events (> 10 mothers)	7	7 (100)	0 (0)
Lactating mothers who attended donor recruitment (total):	762	579 (76)	183 (24)
One-on-one	570	387 (67.9)	183 (32.1)
Group	185	185 (100)	0 (0)
Mothers who expressed interest in donating after recruitment	397	270 (68)	127 (32)
Mothers screened	321	164 (51.1)	157 (48.9)
Lactating mothers screened and who met all requirements to be donors	324	166 (51.2)	158 (48.8)
Eligible human milk donors taught proper hygiene and donation skills ^b^	321	164 (51.1)	157 (48.9)
New donors	314	156 (49.7)	158 (50.3)

*Note*. Reporting period is February 2017–April 2019. Data presented as value (row percentage of the total). ^a^Including donors who were hospital staff who typically expressed milk at home but occasionally in the hospital. ^b^Skills include proper hand washing; safe expression of milk; and how to store, label, and safely handle donor human milk.

**Table 4:** Volume of Donor Milk Received and Pasteurized at the Human Milk Bank at Danang Hospital for Women and Children.

	Location		
	Total Volume	In Hospital Volume (%)	Out of Hospital^a^ Volume (%)
Volume of milk donated (L)	4401	1301 (29.6)	3100 (70.4)
Volume of pasteurized donor milk (PDHM; L)	4173	1268 (30.4)	2906 (69.6)
Passed pre-pasteurization test (L)	1995	722 (36.2)	1273 (63.8)
Passed post-pasteurization test (L)	2869	711 (24.8)	2158 (75.2)
Volume of donor milk disposed (L)	727	332 (45.7)	395 (54.3)
Volume of PDHM at the HMB at the time of reporting (L)	1033	437 (42.3)	596 (57.7)

*Note*. Reporting period is February 2017–April 2019. HMB = human milk bank; PDHM = pasteurized donor human milk. Data presented as value (row percentage of the total). ^a^Including donor milk from hospital staff who expressed milk at home and occasionally in the hospital.

**Table 5:** The Performance Indicators (Use of Pasteurized Donor Human Milk) Grouped by Location.

Performance Indicators	Location		
	*N*	Unit, *n* (%)	Postnatal and Postoperative Unit, *n* (%)
Volume of distributed PDHM (L)	3139	2035 (64.8)	1104 (35.2)
Number of newborns who used PDHM from HMB	8071	2784 (34.5)	5286 (65.5)
Volume of PDHM distributed to infants (L)	3042	1968 (64.7)	1074 (35.3)
Average number of days using PDHM (days)^a^	03	04	01

*Note*. Reporting period is February 2017–April 2019. HMB = human milk bank; PDHM = pasteurized donor human milk. Data presented as value (row percentage of the total). ^a^ Among those who stopped receiving PDHM during the reporting period.

Lactation support was strengthened for all mothers at the hospital by training hospital staff for improved breastfeeding, milk expression, and milk storage practices. Additionally, the availability of the HMB services and access to the HMB facility created additional touch points for lactation support. Overall strengthening of lactation support included key messages for early and frequent breastfeeding or milk expression, promoting the mother to visit and be present as often as possible, the use of mother’s own milk when possible, and the use and cleaning of breast pump parts (when appropriate). Having PDHM from the HMB improved the safety of alternative feeding practices, when compared to unmonitored sharing of maternal milk on the neonatal unit, a practice that is not common everywhere and in which global frequency is not well tracked or understood. Other benefits included: improved breast pump cleaning practices, availability of refrigeration for human milk storage, staff training on lactation support, and consistency and precision in recording newborn feeding practices in the neonatal unit.

#### Continued Quality Improvement

Quality control assessment systems were implemented, using the HACCP protocol and rigorous SOPs. Audit systems were developed for the HMB internal quality assurance team to implement on a quarterly basis. Ongoing quality assurance visits were enacted both by hospital-based teams, project-level teams, and international HMB experts. Quality control visits from PATH and Alive & Thrive were conducted through visits from local project staff and international support. In addition, an international HMB expert from Scotland provided technical assistance on-site for evaluating the use of PDHM and the safety of the HMB processes and strengthening training for breastfeeding and lactation support for mothers with vulnerable infants.

The internal quality assurance team for the HMB included clinical staff representatives from various hospital departments, including neonatal care, and postnatal care, lab and infection control departments, hospital quality control and nursing management units, and the HMB operations staff. Internal auditing for safety and quality HMB integration included: compliance review of the HMB guidelines, maintenance and functionality of the equipment, hygiene and safety of the HMB facility, and staff performance.

## Challenges Faced in Implementation

Challenges faced in the implementation process of the DNHWC’s HMB system for optimal newborn nutrition included the financial and time costs of doing the intervention for the first time in a country, without access to ongoing technical monitoring and support structures or examples in the proximal area. Additionally, the lack of global guidelines for establishing and operating HMBs and the critical need for procedures to be contextually driven made it difficult to create a streamlined approach. The resources required are extensive; however, it does have potential, future cost-saving opportunities due to the improved health outcomes associated with the use of PDHM (Ganapathy et al., 2012; Mahon et al., 2016; Trang et al., 2018). Ongoing costs for HMB operations continue to be challenging at this site. However, they are not beyond what the hospital is able to commit to, and leadership has indicated support for ongoing ownership.

### Expansion of Model and Sharing Results (May 2017 to date)

#### In Vietnam and Southeast Asia

The Danang model has the potential for success in improving neonatal nutrition in other regions within Vietnam. For effective replication of this model to serve multiple regions in Vietnam, a minimum of two to three additional HMBs integrated into facilities have been proposed by the MoH. A regional-based model where the HMB supplied to hospitals serving sick and vulnerable newborns in the region is being developed. The concept is to strengthen the essential programs for lactation support, breastfeeding promotion, and PDHM collection and distribution in the satellite hospitals. Optimal scale-up will require human milk donations to be accepted from external facilities to the hospital, requiring cold-chain systems for safe transportation.

Other settings, including Glasgow, Scotland, have employed this national HMB model successfully to serve all the neonatal care sites in the nation, and hold promise as examples for Vietnam’s success. The MoH has indicated the potential for expansion to hospitals in northern and southern Vietnam. DNHWC is already mentoring future HMBs in Vietnam and across the Southeast Asia region, supported by policy leaders’ strong ongoing commitment and recognition of this HMB as a center of excellence. To date, the DNHWC HMB has hosted five knowledge exchange visits from hospital leadership in Vietnam, China, Indonesia, and Myanmar. The HMB staff has also provided technical assistance to other hospitals in Vietnam and in Southeast Asia. The second HMB in Vietnam recently opened in April 2019, at Tu Du Hospital in Ho Chi Minh City, the largest maternity hospital in Vietnam with 70,000 births each year. As a regional leader, Vietnam serves as a model for improving health systems to support all infants to receive human milk as the primary diet, prioritizing mother’s own milk for her own infant, and providing PDHM for the most vulnerable neonates in need.

#### Global Linkages

The experience of establishing a HMB within the DNHWC context is not unique; other LMIC HMB programs share similar challenges in safety, quality, and the availability of functional support systems (i.e., access to lab facilities, cold chain transportation, and the provision of trained lactation support for all mothers). This first HMB in Vietnam can serve as an example for integrating services and improving neonatal nutrition programs globally. The Vietnam experience was one of many country experiences that informed the development of the Resource Toolkit for Establishing and Integrating Human Milk Bank Programs—A Global Implementation Framework (PATH, 2019a).

## Conclusion

The successful integration of a HMB into the existing health system at DNHWC for improved maternal lactation support and infant feeding practices provides an exemplary model for other hospitals caring for sick and vulnerable newborns in Vietnam, the broader Southeast Asia region, and globally. Several enabling factors and essential program components from this experience are adaptable and applicable to settings seeking to implement new HMB systems or improve existing systems. Utilizing technical expertise from HMB experts globally ensured Vietnam’s first HMB was not built in isolation, but instead built upon over 100 years of international learning in the processing and provision of PDHM through HMBs. Especially in the absence of global guidance and WHO recommendations, learning from experiences globally is vital for the success of future programs. Aligned nutrition and newborn policies at global and regional levels, as well as global guidance, standards, and a network for sharing best practices are needed to improve the safe, quality, and appropriate provision of PDHM to infants in need. A comprehensive approach to integrate HMB systems within newborn and nutrition programming can ensure all infants have equitable access to human milk. Successful implementation of an integrated HMB model in a country or region will help to protect, promote, and support breastfeeding practices which, in turn, will help to reduce infant deaths, especially for the small and sick newborns.
